# Particle Formation by Supercritical Fluid Extraction and Expansion Process

**DOI:** 10.1155/2013/538584

**Published:** 2013-10-07

**Authors:** Sujuan Pan, Junbo Zhou, Haiting Li, Can Quan

**Affiliations:** ^1^Engineering Research Center for Polymer Processing Equipment, Ministry of Education, School of Mechanical and Electrical Engineering, Beijing University of Chemical Technology, Beijing 100029, China; ^2^Division of Chemical Metrology and Analytical Science, National Institute of Metrology, Beijing 100013, China

## Abstract

Supercritical fluid extraction and expansion (SFEE) patented technology combines the advantages of both supercritical fluid extraction (SFE) and rapid expansion of supercritical solution (RESS) with on-line coupling, which makes the nanoparticle formation feasible directly from matrix such as Chinese herbal medicine. Supercritical fluid extraction is a green separation technology, which has been developed for decades and widely applied in traditional Chinese medicines or natural active components. In this paper, a SFEE patented instrument was firstly built up and controlled by LABVIEW work stations. Stearic acid was used to verify the SFEE process at optimized condition; via adjusting the preexpansion pressure and temperature one can get different sizes of particles. Furthermore, stearic acid was purified during the SFEE process with HPLC-ELSD detecting device; purity of stearic acid increased by 19%, and the device can purify stearic acid.

## 1. Introduction

The size of organic and inorganic materials is a key factor that can determine its final use. The well-known conventional processes for particle size redistribution of solid materials are crushing [1] or grinding [2], carried out at cryogenic temperatures for some compounds [3], air micronization, sublimation [4], or recrystallization from solution [5]. There are several practical problems associated with the above-mentioned processes. Some substances are unstable under conventional milling conditions, and in recrystallization processes, the product is contaminated within solvent, and waste solvent streams are produced [6].

Supercritical fluid technology has been widely used for various applications such as extraction, reaction, chromatography and material processing [7, 8]. Several authors have reviewed the applications of supercritical fluid on the preparation of nanomaterials [9]. Carbon dioxide is the most commonly used supercritical fluid owing to its nontoxic, nonflammable, and environmental friendly properties and mild supercritical conditions. Classification and selection of supercritical particle formation processes have been reviewed by several authors [10–12]. Micronization of Stearic acid using supercritical fluid technology involves much simpler steps that can eliminate many disadvantages of the traditional processes [13, 14]. 

On the bases of patent [15] published by Quan from China metrology institute, the author of this paper does research and develops combination device of supercritical fluid extraction and rapid expansion of supercritical solution, which is named as supercritical fluid extraction and expansion (SFEE). SFEE has extraction and granulation functions and can be used separately or combined. Stearic acid is a kind of important industrial raw material, which is widely used in manufacturing cosmetics, plastic hardy plasticizer, release agent, stabilizer, surfactant, rubber vulcanization agent, waterproofing agent, brightener, metallic soap, softener, pharmaceuticals, and other organic chemicals. There are various existing mature ways for stearic acid in industrial production; It is a new attempt and approach to use SFEE technology for getting stearic acid particles. In this study, the feasibility of the SFEE process on the micronization of stearic acid was investigated. Examinations of the solid-state properties such as the particle size and its distribution, as well as the purity change of stearic acid after processing by SFEE, that is, stearic acid solubility in the CO_2_ SCF, are demonstrated.

## 2. Experimental Method

### 2.1. Materials

Stearic acid granules (C18:0FFA) with a mean particle diameter of approximately 4.5 mm with purity about 60% was supplied by Qinghongfu Technology Co. (Beijing China). Liquid carbon dioxide with purity of 99.9% used for SFEE process was purchased from Praxair Gas Co. (Beijing, China).

### 2.2. Apparatus and Procedure

**Figure 1:**
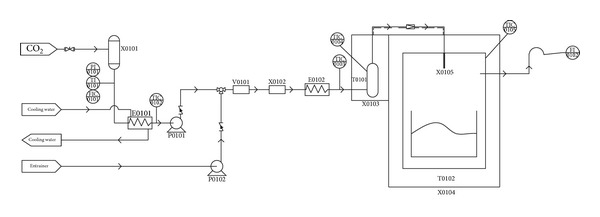
Process flow diagram. X0101 purifier; E0101 heat exchanger; P0101 CO_2_ pump; P0102 entrainer pump; V0101 mixer; X0102 purifier; E0102 heat exchanger; X0103 incubator; X0104 incubator; T0101 extraction oven; T0102 expansion oven; X0105 nozzle.

The schematic diagram of the SFEE process is shown in Figure 1. The experimental apparatus in this paper was developed by the research team, mainly five parts included: pressure control unit, temperature control unit, injection unit, extraction unit, and expansion unit. Prior to the start of the experiment, three temperatures of temperature control unit should be set: pump cooling unit (purchased from Hengping instrument, Shanghai) adjusted to 276 K and extraction unit and expansion unit temperature set to the required temperature. Preextraction temperature (extraction oven temperature) and expansion temperature (expansion oven temperature) are heated controlled by incubator (our team developed it with Beijing Luxi technology together), which is formed from two chambers, resulting in temperature, respectively, controlled, without interference. 

SCF-CO_2_ pressure is mainly provided by supercritical special high-pressure pump (supplied by Thar), which is controlled by the back pressure valve (supplied by Tescom). The resolutions for the measurements of preexpansion temperature and preexpansion pressure were ±0.1 K and ±0.01 MPa, respectively. SCF-CO_2_ first obtains stable flow rate and pressure through cooling unit and supercritical pump then goes into extraction kettle (500 mL, maximum pressure 500 MPa, Thar), 10 g stearic acid should be put into the extraction kettle in advance, statical SFEE process can be performed for certain time with target components dissolve. Afterwards, open back pressure valve; high pressure SCF-CO_2_ dissolved with target component would pass through the nozzle (supplied by Thar) to a container under atmospheric environment, at this moment RESS process is completed. Stearic acid powder should be collected and put on a substrate, which will be used for further analysis and research later on. 

### 2.3. Particle Characterization

Particle size and morphology of the sample specimen were analyzed by scanning electron microscopy (JSM-6700F, JEOL) after coating with a thin gold/palladium film with the aid of a sputter coater SC7640 (Quorum Technologies, UK).

### 2.4. Particle Size Distribution

The particle size distribution was obtained according to the following procedure. Firstly, 100 randomly selected, well-separated particles from the SEM image were measured in zoom-in mode, in which individual particles can be recognized clearly, using MATLAB. The shortest distance observed across each particle was taken, here called particle diameter for simplicity. Secondly, the particle sizes were calculated based on the ratio of their diameters to the SEM magnification scale in the normal mode in MATLAB. Finally, a particle size distribution histogram was drawn, and the mean particle size diameter, *a* (*μ*m), and the standard deviation, *d* (*μ*m), in normal distribution mode *N*(*a*, *d*) were estimated by MATLAB functions.

### 2.5. Purity Test

Stearic acid samples and products are tested by HPLC-ELSD separately, by comparing its purity before and after the change, which can indirectly verify the solubility of stearic acid in SCF-CO_2_. Chromatographic column is BDS HYPERSIL C8 150 mm × 4.6 mm (Thermo, US), the detector is Evaporative Light-Scattering Detector (ELSD, SEDERE, France), and chromatography condition is with column temperature 308 K, flow rate of 1 mL/min, mobile phase acetonitrile to water (85 : 15, *φ*), isocratic elution, ELSD parameters with drift tube temperature 308 K, gain 7, and pressure 0.35 MPa. Quantitative adopts standard substance external standard method.

## 3. Results and Discussion

### 3.1. Effect of the SFEE Process Parameters

In RESS, over saturation of solute *S* is a very important parameter [16] as follows:

(1) $$\begin{array}{c} S=\frac{\varphi \left(T,P,y_{E}\right)y_{E}\left(T_{E},P_{E}\right)}{\varphi \left(T,P,y^{*}\right)y^{*}\left(T,P\right)}, \end{array}$$

where, *φ*(*T*, *P*, *y*_*E*_) is the fugacity coefficient (dimensionless) when solute exists in real mixture on the condition of temperature *T*, pressure *P*, and concentration *y*_*E*_. *y*_*E*_(*T*_*E*_, *P*_*E*_) is the solute molar fraction (dimension: 1) under extraction temperature and extraction pressure.  y*(*T*, *P*) is the mole fraction of solute (dimension: 1) under temperature *T* and pressure *P*.

According to the classical nucleation theory [17] and RESS research on nuclear theory of Türk [18, 19], the two most important parameters in the RESS method are critical nucleus radius γ* and the critical concentration of the nuclei N*, the formula is as follows:

(2) $$\begin{array}{c} \gamma^{*}=\frac{2\sigma v_{2}^{s}}{\kappa T_{pe}\left(\ln S\right)}, \end{array}$$

(3) $$\begin{array}{c} N^{*}=N_{2}\exp\left(-\frac{16\pi }{3}\left(\frac{\sigma {\left(v_{2}^{s}\right)}^{2/3}}{\kappa T_{pe}}\right)\left(\frac{1}{\ln S}\right)\right), \end{array}$$

where, *σ* is the surface tension (N/m) between two phases of solid-liquid,  *v*_2_^*s*^ is solute molecular volume (cm^3^/mol), *κ* is Boltzmann's constant (J/K),  *T*_*pe*_ is the critical temperature (K), and  *N*_2_ is the number of liquid solvent concentration (cm^−3^).

Two most important parameters about RESS can be obtained from the above formulae: preexpansion pressure and the preexpansion temperature. Increasing the preexpansion pressure can increase the concentration of the critical nucleus and over saturation, and reduce the radius of the critical nucleus conducive, which is benefit for RESS experiment to a better direction (Nucleation small, uniform distribution). Surely, the preexpansion pressure cannot increase indefinitely, which will increase the investment in equipment and increase the risk of experiments required to seek the best value. The impact of preexpansion temperature involves two aspects: the increase of temperature will reduce the concentration of SCF which means the decrease of solubility, while the saturated vapor pressure of the solute will be enhanced; we can see from formulae (2) and (3) that low temperature means the increase of critical number of nuclei, which is advantageous to the experiment.

The effects of operation parameters in the SFE and RESS process had been studied in the literature usually by the method of changing one factor at a time. That method is simple but might neglect any possible cross-interaction between operation parameters. This disadvantage could be improved by applying the method of design of experiment. In our SFEE experiments, a full four factors and two-level factorial design was adopted, and 16 experimental conditions were investigated.

### 3.2. Effect of Preexpansion Pressure

**Figure 2:**
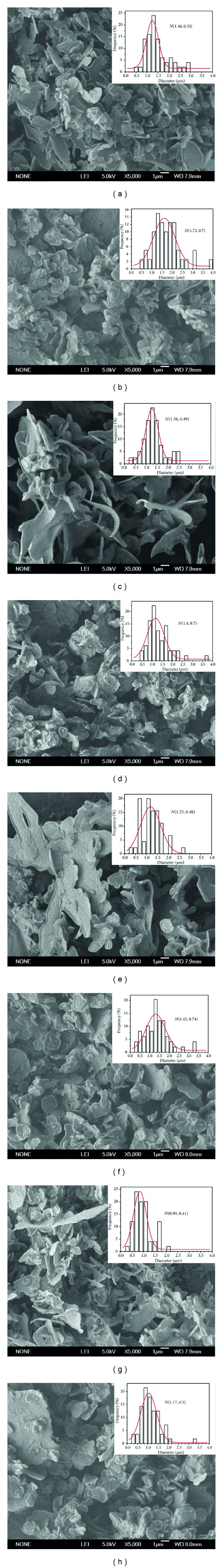
SEM micrographs of stearic acid particles collected at different conditions: (a) 15 MPa, 333 K; (b) 15 MPa, 348 K; (c) 20 MPa, 333 K; (d) 20 MPa, 348 K; (e) 30 MPa, 333 K; (f) 30 MPa, 348 K; (g) 35 MPa, 333 K; (h) 35 MPa, 348 K.

In order to study the effect of expansion pressure on stearic acid particles, four different groups of pressure experiments were operated (15, 20, 30, and 35 MPa); at the mean time, preexpansion and expansion temperature remain unchanged at 333 K and 348 K. Product SEM figures, average particle size distribution histogram, and standard variance diagram are shown in Figure 2. 

According to the formulae (1), (2), and (3), when increasing the preexpansion pressure (keep the preexpansion temperature as constant), the over saturation of solute *S* and the critical concentration of the nuclei N* will increase obviously and reduce the critical nucleus radius γ*. The comprehensive effect of the three parameters is to reduce the particle radius and tend to be more uniform distribution.

Comparing Figures 2(a), 2(c), 2(e), and 2(g), you can find at temperature 333 K, the average particle size of stearic acid reduces from 1.46 *μ*m to 0.99 *μ*m, with the pressure rising from 15 MPa to 35 MPa. Comparing Figures 2(b), 2(d), 2(f), and 2(h), you can see when the temperature is at 348 K, the average particle size of stearic acid reduces from 1.7 *μ*m to 1.17 *μ*m, with the pressure rising from 15 MPa to 35 MPa. From above it can be seen that in stearic acid SFEE experiment, the average particle size is decreased with the increase of pressure. 

### 3.3. Effect of Preexpansion Temperature

In order to study the effect of preexpansion temperature on stearic acid particles, a series of four experiments under different temperatures and pressures are also conducted. Products SEM figure, average particle size distribution histogram, and standard variance diagram are shown in Figure 2. In this experiment, preexpansion pressure is fixed in order to compare the preexpansion temperature. 

As above, the impact of the preexpansion on particulate properties is multifaceted. On one hand, the increase of temperature can improve the saturated vapor pressure of the solute; on the other hand, the high temperature also reduces the density of the supercritical fluid, namely, reduce the solubility of the solute. But we can conclude merely from formula (3) that the low temperature will increase the concentration of the critical nucleus which will be conducive to the precipitation of substances.

Comparing Figures 2(a) and 2(b), 15 MPa pressure keeps constant, as the temperature is increased to 333 K from 348 K, and the average particle size of product increases from 1.46 *μ*m to 1.72 *μ*m. Comparing Figures 2(c) and 2(d), pressure maintains 20 MPa, with the temperature increasing from 333 K to 348 K, and the average particle size of product increase from 1.36 *μ*m to 1.4 *μ*m. Comparing Figures 2(e) and 2(f), pressure maintains 30 MPa, with the temperature increasing from 333 K to 348 K, and the average particle size of product increases from 1.25 *μ*m to 1.45 *μ*m. Comparing Figures 2(g) and 2(h), pressure maintains 35 MPa, with the temperature increasing from 333 K to 348 K, and the average particle size of product increases from 0.99 *μ*m to 1.17 *μ*m. Thus it can be seen that increasing preexpansion temperatures is unfavorable to reduce the average particle size of the products. 

### 3.4. Stearic Acid Purity Characterization

The measurement methods for stearic acid purity are various; gas chromatography is mostly adopted, and however, the method needs to reduce the boiling point of stearic acid by esterification reaction prior to the intake phase. Because esterification reaction is reversible reaction, it cannot guarantee completion rate, so the purity of gas phase measured is not the most accurate (comparing is feasible, of course). That is why in this paper, raw materials and products purity is tested through high performance liquid chromatography (HPLC-ELSD).

Chromatographic column is BDS HYPERSIL C8 150 mm × 4.6 mm; chromatography condition are with column temperature 308 K, flow rate of 1 mL/min, mobile phase acetonitrile to water (85 : 15, *φ*), isocratic elution, ELSD parameters with drift tube temperature 308 K, gain 7, and pressure 0.35 MPa. The quantitative standard is external standard method. The quantitative standard is substance external standard method. The formula of calculating the purity by external standard method can be seen in (4). All test results of samples are shown in Table 1.

Consider

(4) $$\begin{array}{c} p_{2}=\frac{c_{1}}{c_{2}}\frac{A_{2}}{A_{1}}p_{1}, \end{array}$$

where, *p*_1_, *p*_2_ represent the purity of the standards and the sample, *c*_1_, *c*_2_ represent the concentration of the solution of standard and test sample, and *A*_1_, *A*_2_ represent the peak area of the standard and the sample.

**Figure 3:**
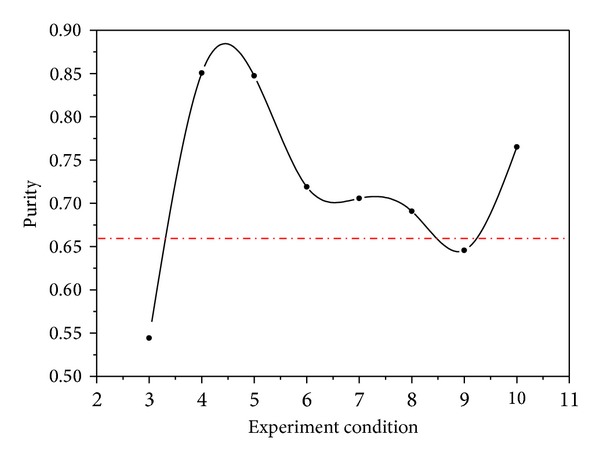
Stearic acid purity trend chart (serial number meaning with Table 1).

The picture of stearic acid purity trend can be seen in Figure 3, which has the same meaning as Table 1. The dotted line is the purity of stearic acid in the raw material. The purity is under the dotted line, which means that the solubility of stearate is so small that it does not have a purification effect on raw material. In the area of low pressure, the solubility of stearic acid increased with the increase of pressure, and the greatest value appears between 4 and 5. It means that during this area, purifying stearic acid is meaningful. In further research, the orthogonal test pressure level values should be within this range 20–30 MPa. After the maximum value, the solubility of stearic acid began to decline, first rapidly then it slowed down. After 9, it increases, but the pressure then is over 30 MPa, and the temperature is 348 K, which means high cost of experiment, relatively speaking.

Through this experiment, the impact of pressure and temperature on stearic acid in SCF CO_2_ can be obviously derived: with the increase of pressure, the solubility of the stearic acid is gradually increasing, like points 4 and 5; peak area gradually increased to the maximum value, but the solubility decreases with the increase of temperature, like points 6 and 7; the peak area decreases gradually, even lower than the containing of raw materials. So the solubility of stearic acid in the SCF-CO_2_ will increase with the increase of pressure and decrease with the increase of temperature. Considering the two factors, and taking into account the factors of safety and cost of experiments, it is better to extract between 20–30 MPa and low temperature region (333 K).

## 4. Conclusions

1. We developed experimental device using supercritical fluid extraction and expansion (SFEE) technology and prepared the stearic acid submicron particles by this apparatus with particle size of 0.9~2 *μ*m. The size of stearic acid particles under the conditions of 35 MPa and 333 K is 0.9 *μ*m, a sub-micron level. Smaller sized particles can be obtained by adjusting the experimental parameters and nozzle size.
2. The higher the pressure of the preexpansion is, the smaller the average particle diameter will be, and the higher the preexpansion temperature is, the smaller the stearic acid particle will be. Different sizes of particles can be achieved by adjusting the pressure of the preexpansion and preexpansion temperature. Stearic acid can be extracted in SFC-CO_2_ by this apparatus which realizes the extraction functions.

## Figures and Tables

**Table 1:** Test results of stearic acid by HPLC-ELSD.

No.	Experimental condition	Peak time (min)	Peak area	Purity (%)
1	Standard substance	5.4	12325	99.8
2	Raw material	5.1	8169.3	66.1
3	SFEE, 15 MPa, 333 K	5.1	6721.4	54.4
4	SFEE, 20 MPa, 333 K	5.2	10504.8	85.1
5	SFEE, 30 MPa, 333 K	5.1	10463.5	84.7
6	SFEE, 35 MPa, 333 K	5.1	8879.6	71.9
7	SFEE, 15 MPa, 348 K	5.1	8715	70.6
8	SFEE, 20 MPa, 348 K	5.1	8530.6	69.1
9	SFEE, 30 MPa, 348 K	5.1	7973.9	64.6
10	SFEE, 35 MPa, 348 K	5.1	9449.2	76.5
